# CAP-T cell expression system: a novel rapid and versatile human cell expression system for fast and high yield transient protein expression

**DOI:** 10.1186/1753-6561-5-S8-P133

**Published:** 2011-11-22

**Authors:** Jens Wölfel, Ruth Essers, Corinna Bialek, Sabine Hertel, Nadine Scholz-Neumann, Gudrun Schiedner

**Affiliations:** 1 CEVEC Pharmaceuticals GmbH, Cologne, Germany

## Backround

CAP (CEVEC's Amniocyte Production) cells are an immortalized cell line based on primary human amniocytes. They were generated by transfection of these primary cells with a vector containing the functions E1 and pIX of adenovirus 5. CAP cells allow for competitive stable production of recombinant proteins with excellent biologic activity and therapeutic efficacy as a result of authentic human posttranslational modification. In order to gain access to the benefits of the CAP technology also for early research, target evaluation or assay development, the transient expression system CAP-T was developed. CAP-T cells are based on the original CAP cells and additionally express the large T antigen of simian virus 40 (SV40).

## Results

**Figure 1:**
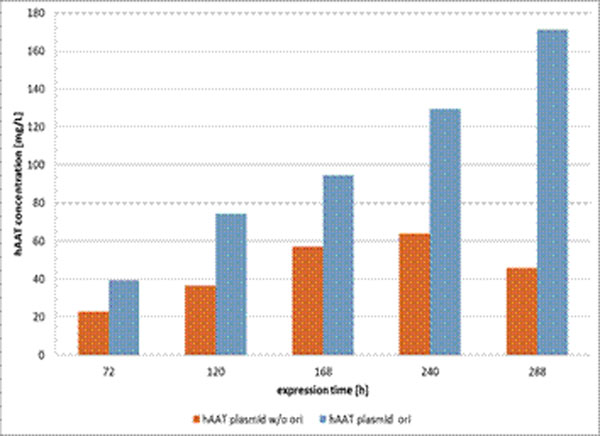
Transient expression of hAAT in CAP-T: Two plasmids containing a hAAt expression cassette were transfected by nucleofection (1 x 10^7^ cells). The plasmid containing a SV40ori (ori) yielded about 2.5 time higher expression levels at maximum product concentration than a plasmid lacking this ori (w/o ori).

To characterize the CAP-T expression system, they were transiently transfected with an expression plasmid for the highly complex and glycosylated human α1-Antitrypsin (hAAT). This resulted in remarkably high expression levels of up to 60 mg/L. These levels could be even increased 2.5 fold by adding an SV40 origin of replication (SV40ori) to the expression vector (Figure 1). When compared to HEK293T cells, CAP cells showed a significantly higher expression titer than HEK293T cells (data not shown). In order to understand these phenomena, the copy number of the expression plasmid, upon transfection, was determined. CAP-T efficiently replicated the expression plasmid containing the SV40ori, resulting in increasing copy numbers per cell over time yielding in about 4 times higher copy numbers than in HEK293T cells transfected with the same plasmid and in CAP-T cells transfected with the plasmid lacking the SV40ori (data not shown) and consequently in higher expression levels. In order to establish a more scalable transfection method than nucleofection, the suitability of different transfection reagents for the transfection of CAP-T cells was determined. In these experiments beside nucleofection, 293fectin and polyethylenimin (PEI) based transfection methods showed best results in small scale transfections, enabling the scalability of the transient transfection in CAP-T cells (data not shown). With the PEI based transfection method hAAT could be produced also in 300 mL shaking culture and 1L bioreactor with product titers of up to 180 mg/L in simple batch processes of up to 10 days (data not shown). Several other glycosylated proteins have been tested in transient transfections of CAP-T cells yielding comparable product titers (Table 1).

**Table 1:** Table 1 summarizes the relevant data from transient transfections of CAP-T cells in different scales with plasmids coding for different proteins of interest (hAAT, erythropoietin (EPO), C1-Inhibitor and IgG). Cells were either transfected by nucleofection (1 x 10^7^ cells) or by PEI (1.7 x 10^9^ cells).

	hAAT		EPO	C1-Inhibitor	IgG
cells transfected	1 x 10^7^	1.7 x 10^9^	1 x 10^7^	1 x 10^7^	1 x 10^7^
culture volume [ml]	30	1000	60	30	30
culture time [days]	9	6	10	12	6
viability at harvest [%]	85	70	80	87	80
**volumetric productivity [mg/L]**	**170**	**180**	**38**	**35**	**150**

## Summary

In summary, CAP-T cells present a highly efficient transient expression system enabling the generation of mg amounts of the protein of interest for early research and development within only two weeks from gene to product. Furthermore, CAP-T cell produced proteins showed fully human posttranslational modification pattern, which was also observed for the original human CAP cells, the CAP-T cells were derived from. The CAP technology based on CAP-T cells for transient transfection and CAP cells for stable protein production [1] therefore provides a unique system in which the whole process from early research to production of therapeutic proteins can be run through with the same cell type.
